# A Mixed Method Research to Identify Perceived Reasons and Solutions for Low Uptake of Cervical Cancer Screening in Urban Families of Bhopal Region

**DOI:** 10.1155/2016/5731627

**Published:** 2016-04-12

**Authors:** Nancy Jain, Ajay Halder, Ragini Mehrotra

**Affiliations:** All India Institute of Medical Sciences Bhopal, Madhya Pradesh 462022, India

## Abstract

Low uptake of cervical cancer screening is not a matter of poor coverage of health care facilities only. We wish to identify the perceived reasons behind low uptake of screening in Bhopal region and also possible solutions for an urban setting. In a mixed research, through a series of focused group discussions, we wished to do thematic interpretation of the perceptions towards cervical cancer screening by deductive content analysis of FGD and also to obtain a free list of perceived causes and solutions with Smith's saliency score and perform cluster analysis by pile sorting. We found that the perceived reasons could be grouped into three themes which were* (1) information gap leading to fear of unknown, (2) casual attitude*, and* (3) resource constrains and affordability issues*. For the perceived solutions there were 11 codes which could be grouped into two groups; these were* increasing awareness* and* vaccination*. Free list of perceived reasons and solutions has also been generated. No single solution can be suggested but a comprehensive approach with awareness campaigns, personalized encouragements, affordable and friendly health care with subsidized vaccination, and screening facilities are expected to increase awareness and acceptability and thus reduce burden of disease in the long run.

## 1. Introduction

Cervical cancer is the third most commonly diagnosed cancer and the fourth leading cause of cancer death in women worldwide [1]. Estimated 529,000 new cases and 275,000 deaths occurred in 2008 out of which 79–83% of new cases were diagnosed in developing countries [2], whereas, in developed countries, cervical cancer accounts for only 3.6% of new cancers, with a cumulative risk of 0.8% (age 0 to 64 years) [3].

Cervical cancer is the most common type of cancer in Indian females aged 15 years and above [1]. With a lifetime incidence of 1 : 53 (versus 1 : 100 developed regions) 74,000 new cases were diagnosed in 2010 [4]. Since 70% cases are diagnosed at stage III or IV there is 32% mortality rate, 30% 5-year survival for stage III, and a dismal 6% for stage IV. In an estimate there will be 225,000 new cases in 2025 [5].

Screening of adult women for cervical cancer and adolescent HPV vaccination can prevent two out of three cervical cancer deaths [4]. In contrast to high-income countries, where cervical cancer screening is offered as part of routine primary care, few large-scale screening programs exist in India [5]. Moreover, though primary prevention through human papilloma virus (HPV) vaccination is gaining acceptance in high-income countries and has been endorsed by the World Health Organization (WHO), vaccine awareness, access, and use are very low in India [6, 7]. Most of the cervical cancer screening in India is opportunistic and almost negligible voluntary screening. The District Cancer Control Program initiated in year 2005 under the National Cancer Control Policy of Ministry of Health and Family Welfare, Government of India, strives to achieve larger coverage of women with early detection and screening through the exiting health care system at the district level free of cost [8]. But large-scale community level cervical cancer screening program outside research settings is nonexisting in India at present. Guidelines for cervical cancer screening in India were issued in 2005 [9] which address the two basic challenges in achieving wider cervical cancer screening coverage in India. Firstly it outlines the methods of community sensitization and motivation to achieve universal screening and secondly it advices the use of visual inspection methods (visual inspection under Lugol's iodine, VILI; visual inspection under acetic acid, VIA) instead of cytology to curb the cost and the need for repeated hospital visits. But lack of awareness, fear of diagnosis, shying from pelvic examination, and so forth are important causes which keep even urban educated women away from voluntary cancer screening. Over the years with research and deliberation in this field it is now clear that low uptake of cervical cancer screening not only is a matter of poor coverage of health care facilities but also is equally or more importantly due to negative attitude of women themselves, their families, and to some extent a general behavior of neglect on the part of service providers [10]. Consequently it becomes prudent to bring about change in the perceptions of the stake holders regarding importance of cervical cancer screening and vaccination.

Through this research, we wish to identify the perceived reasons behind low uptake of screening among a sample of urban population and their health care providers in Bhopal region and also identify possible solutions for an urban setting.

The aims and objectives of the study can be summarized as follows:

1. thematic interpretation of the perceptions of the women and health care providers towards cervical cancer screening by deductive content analysis of focused group discussion (FGD);
2. obtaining a free list of perceived causes and solutions of low uptake of cervical cancer screening among women and their health care providers with Smith's saliency score;
3. making groups of the above listed perceived causes and solutions with high Smith's saliency score (cluster analysis);
4. obtaining the consensus measure (%) among women about various issues raised in the questionnaire.

## 2. Material and Methods

### 2.1. Study Setting

The study is a community based cross-sectional mixed method research with both quantitative and qualitative methods. For qualitative study we conducted focused group discussion of stake holders followed by* deductive content analysis* [11]. And for the quantitative part we assessed the* knowledge, attitude, and practice* of women through a structured questionnaire. To reach out to the target of cervical cancer screening, study was undertaken at community level in various blocks of Bhopal which is the capital city of Madhya Pradesh.

### 2.2. Participants

In a mixed research, questionnaire based interviews (quantitative method) and focused group discussion (qualitative method) were used to understand the perceived barriers to cervical cancer screeching in the urban population of Bhopal [12]. Participants were women aged between thirty and sixty years from urban residential areas and slums and health care professionals who can possibly encourage and administer cervical cancer screening like gynecologist and general practitioners.

### 2.3. Sampling Method and Sample Size

For the questionnaire, multistep cluster sampling was done and the sample size was the number of items in the questionnaire × 10.

For the qualitative research methods we use nonprobability sampling method; in this case we used purposive sampling method. Sample size was taken as the number of cases till saturation of responses that is where it stops yielding further new information [13].

### 2.4. Tool Development

A questionnaire verifying the* knowledge, attitude, and practice* of the women about present scenario of cervical cancer screening was developed by the researcher with dichotomous response (yes or no). After key informant interview with five gynecologists and 2 women eligible for cervical cancer screening a draft questionnaire was developed with 28 items. It was face validated by the experts of the fields like experienced gynecologists and epidemiologist. Eight items were removed for poor content validity and wording of few items was changed for ease of interpretation. Finally the tool contained 20 items with six items representing the demographic data with Kuppuswamy socioeconomic class. Thirteen items verified the “*knowledge*” of women towards cervical cancer, its risk factors, and vaccination. Each item was given a weightage according to its subjective importance for the laywomen from minimum of 1 to a maximum weightage of 5. The total score therefore could vary from a maximum of 38 (100%) and a minimum of 0 (0%). A score of ≥19 (≥50%) was considered “*adequate”* and women with scores ≥ 29 (≥75%) were considered “*well aware.”* The “*practice”* was assessed by the percentage of women who underwent cervical cancer screening. Before final administration the questionnaire was pilot tested on a small sample of women attending the gynecology outpatient departments in AIIMS Bhopal Hospital to identify ambiguous wording and double barreled questions.

### 2.5. Data Collection

Women with age between 30 and 60 years were approached in a door to door survey by interviewers in four wards of Bhopal city including two organized residential areas and two slums. After taking informed consent, women were requested to fill survey questionnaire developed for the purpose. A purposive sampling of women who participated in the survey was done and they were invited for* focused group discussion (FGD)* in a convenient time and place in the locality itself. FGD was conducted face to face by an interviewer trained in qualitative research. A group of 6 to 8 willing women were asked questions as per interview guide. A written consent was taken by all participants. The purpose of the study, the procedure to be followed, and its implications were explained to all participants. Proceedings were recorded on audio recorder enabled mobile phones. A transcript was prepared by the interviewer soon after finishing the FGD and cross-checked with the participants for approval. Five such FGDs were conducted with laywomen and four FGDs were conducted with health care professionals including gynecologist, medical graduates, and nursing staff. FGDs with different participants were conducted till the saturation of responses was met. Also a free listing exercise was done to identify major causes of low cervical cancer screening and possible solutions with all the FGD participants. Smith's *S* (Smith's saliency score) refers to the importance, representativeness, or prominence of items to individuals or to the group and is measured in three ways: word frequency across lists, word rank within lists, and a combination of these two [14]. Reasons with relatively high Smith's *S* value were then pile sorted by equal number of participants for cluster analysis.

### 2.6. Data Analysis

The quantitative data will be analyzed using Microsoft Excel 2007*™* (Washington, USA).

A summative approach to qualitative content analysis was undertaken to identify and quantify themes from the text data and infer meaning in the given context [15]. The method suggested by Graneheim and Lundman [16] was adopted. The procedure involved document preparation, open coding, grouping, categorization, and theme abstraction. Since the basic outline of the outcome on the study was known due to previous studies a* deductive content analysis* method was used. The units of analysis were women's and health care professional's individual statements. Statements with similar meaning were grouped together until a point was reached where further collapsing resulted in no loss of qualitatively important information. The data were classified and quantified as simple nonhierarchical typology of various for and against perceptions.

A multidimensional scaling and hierarchical cluster analysis was done with pile sort data to get collective picture of perceived rationale behind “causes” which the women and health care professionals felt went together. The analysis of free list and pile sort data was undertaken using Anthropac 4.98.1/X software [17].

### 2.7. Ethical Issues

This study was sponsored by the Indian Council for Medical Research (ICMR) through its Short Term Studentship (STS) program. All the participants of questionnaire survey were provided with the information and verbal consent was taken. Written consent was taken for FGD participants. The information sheet was read out to those who could not read it. None of the participants' identities was revealed in this study report. This study has been approved by the Institutional Human Ethics Committee, All India Institute of Medical Sciences Bhopal with the Project code STS-0048-2015.

## 3. Results

### 3.1. The Knowledge Attitude and Practice Survey

There were total 306 women who participated in the questionnaire door to door survey. Sixty percent of women were below forty years of age, 95% were married, 65% had education above matriculation, 40% were at least graduated, and 11% were illiterate.  Table 1 shows the demographic details of the participants.

The knowledge score based on the survey questionnaire is presented in Table 2. The median score was 18 with 10 and 26 as the 25th and 75th percentile. Twenty-one percent of women were well informed with score equal to or above 29 (75%), 25% of women were adequately informed, and 54% of women were inadequately informed.

The results of the survey are shown in Tables 3 and 4. Regarding the baseline knowledge 87 percent of women reported that they have heard of cervical cancer. Fifty-six percent of women know that it is the most common cancer affecting women. Fifty percent of women thought that this cancer can affect anyone. But only 38 percent of women could identify at least one common symptom of cervical cancer. Options given were* abnormal vaginal discharge, abnormal vaginal bleeding, bleeding after sexual intercourse*, and* bleeding after menopause*. Only 24% of women could appreciate that this can be caused by sexually transmitted infections and only 40% of women knew that* early age at marriage* or* child birth* could lead to increased risk of cervical cancer. Although 47% of women were aware that cervical cancer is preventable, only 34% of women had actually heard of something called screening for cervical cancer. Only 22% of women knew any centre where cervical cancer testing could be done. Only 11 percent of women reported to have undergone cervical cancer screening. Twelve percent of women knew that a vaccine existed against cervical cancer. At the present cost 30% women agreed that they would get themselves and their family vaccinated but 45% women were not sure as to whether they will be able to get the vaccination done in the present price.

### 3.2. Results of the Analysis of Focused Group Discussion

**Figure 1:**
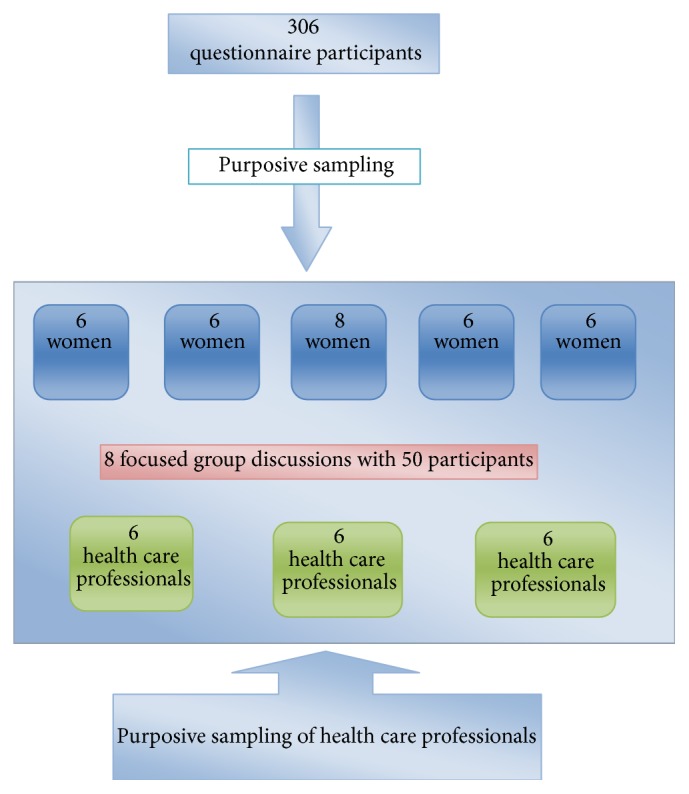
Scheme of recruitment of participants.

There were eight separate focused group discussions of different locations, five with laywomen and three with health care professionals. There were total of 50 participants with 32 women and 18 health care professionals.  Figure 1 shows the scheme of distribution of participants. There were nineteen codes generated for the baseline knowledge and perceptions regarding cervical cancer, its symptoms, and risk factors. There were 23 codes generated for the perceived reasons for low uptake of cancer screening. These codes were grouped into three themes which were* (1) information gap leading to fear of unknown, (2) casual attitude*, and* (3) resource constrains and affordability issues* each containing eight, five, and ten codes, respectively. For the perceived solutions there were 11 codes which were grouped into two groups; these were increasing awareness and vaccination with five codes each.  Table 5 shows the thematic analysis of the focused group discussions and the categorization matrix.

*Content Analysis of Focused Group Discussion*. The following are the category, subcategory, and code wise content analysis which are numbered in the paragraph separated by dots. Same numbers could be referred to in Table 5 for better understanding.

(1.1.1) Women are of the general view that there is gross lack of awareness about cervical cancer. They perceived it to be the most common cancer among women along with breast cancer. The general perception is that women should undergo screening regularly as it is a preventable disease. If treatment is delayed the outcome is more dreaded.

(1.2.1) Regarding the causation of cervical cancer women perceived white vaginal discharge, heavy and irregular bleeding, pain, and itching in private parts to be important symptoms of cervical cancer. Interestingly women were well aware of the fact that sexually transmitted diseases involving uterus and related structures caused cervical cancer. In this effect many emphasized that use of condoms can prevent cervical cancer. Tobacco consumption is perceived as a causative factor.

(1.2.2) Author noted that women perceived abdominal mass including fibroids and ovarian cysts to be important causes of cervical cancer. Many said that decreased bleeding may lead to collection of blood inside the uterus and in turn cause cancer. Unhygienic practices like using dirty linen as sanitary pads during menstruation by young girls of poorer section can cause cervical cancer. Other misconceptions perceived by women to be a causative factor in cervical cancer were surgical abortions and prolapsed uterus and even not wearing undergarments was also thought to be a predisposing factor to infection and thus cervical cancer.

(2.1.1) It was strongly felt that there is lack of awareness about cervical cancer especially among women from rural areas. Illiteracy was the principle cause of lack of awareness and the desire to know about health related issues and opportunities. This leads to fear of cancer, fear of diagnosis, and fear of procedures or surgeries. No effort is made on the part of government to spread awareness about cervical cancer in particular. Since it is a common life-threatening disease more efforts should be made by the government in its prevention. For example, polio could be eradicated only because of widespread awareness and door to door availability of vaccination.

(2.1.2) It is perceived by the participants that women in general lack desire to give importance to personal health related issues. They take their health related issues casually and deny the fact that they can ever get the disease. They even neglect early symptoms and postpone the treatment till it is late. They find themselves busy with family chores most of the time. A lot depends on the family members like husband, mother-in-law, and father-in-law as their concurrence is desirable before any attempt is made to get medical advice.

(2.1.3) When asked to share their experiences and perceptions about health care facilities women unanimously expressed their unhappiness over the lack of facilities, staff, and infrastructure in government hospitals. Long waiting queue and lengthy procedural delays are common. Bad behavior of doctors and nurses add to the agony. Lack of punctuality is also perceived as an important reason of trouble women face in government hospitals. Some women expressed that the level of satisfaction felt after visiting a government facility is poor and also the diagnosis and treatment provided by doctors are unreliable. Although women feel satisfied after visiting private facilities they also emphasize that high cost of treatment precludes its use because most of the people in India are poor. Few women said that private hospital do hysterectomy at a trivial complaint without giving any option for cancer screening or any information about it. They felt presence of male doctors to be a cause of hesitation to visit hospital facilities particularly for examination of private parts. But once they have a problem or surgery is required they do not hesitate to visit male doctors. Some women expressed concern about nonavailability of proper transport to large government hospitals. It takes a lot of money to use private vehicles to reach there, said one woman. They suggested that they would go to camps by government doctors if held in nearby places.

(3.1.1) When asked about the most effective ways of increasing awareness about cervical cancer women expressed that they have been exposed to home to home surveys and seminars by nearby nursing college students regarding various health related issues. And they found it to be enriching and effective. Therefore, they perceived home visits and seminars by health professionals as one of the most effective methods. Moreover they also felt that unless the information is imparted by doctors or other related health professionals they ought to be trivialized and undermined. For medical students they felt the students are actually taking their help in their studies but they do not mind talking to them as women themselves also benefit from it.

(3.1.2) Television was perceived to be another important method which could increase the awareness. This is the most effective way for illiterate women who cannot read newspapers or magazines and write. Moreover television sets are available in many households even in rural areas. Short films and educational advertisements in between television serials will be very effective. It is not only informative to them but also useful for them to convince their husbands for taking up measures. Short films during interval in film theaters will be useful in educating youngsters.

(3.1.3) Information notes on newspapers, magazines, hoardings, and paper pamphlets were suggested by women to be important methods. Slogans and information printed on important documents like rail and bus tickets and so forth could be readily assessed and read.

(3.1.4) Women felt that they can gain from discussion themselves. A group of women said they gathered for yoga in a community centre where they discuss several issues including health related issues. They gain from each other's knowledge. Women expressed that ASHA or Anganwadi workers could participate in such group discussions and increase awareness of the women of the locality. They can alleviate their anxiety about the procedures to be undertaken, cost implications, and so forth.

(3.1.5) Women expressed their unhappiness over lack of attention given to them by doctors when they visit hospitals. They considered the information imparted by the doctors during medical visits to be very useful and effective in behavior change. Women wished that the problem of crowded hospitals and insufficient staff should not come in their way in getting full attention from their doctors.

(3.2.1) The general expression of women regarding the HPV vaccine is that they “never heard about” or “they heard something but not sure what it is” when the vaccine and its efficacy are explained to them; they said they “would like to accept if made available” although they felt worried about the high cost. They expressed their concern that with the present cost the poor will never be able to get their girls vaccinated, more so if a family has more than one girl. If the cost of the vaccine is reduced they would want their daughters to be vaccinated.

*Results of Free Listing and Cluster Analysis*. In order to bring more objectivity and reliability the free listing exercise was done for the causes of low uptake and their perceived solutions. There were seventeen participants in the free listing exercise. Each of them enlisted a set of 2 to 6 causes which they thought were important in the decreasing order of significance. “*Lack of information about cancer*” and “*feel shy to discuss*” emerged as the most commonly perceived barriers. Others were “no attention to personal problems,” “*high cost of treatment*,” and “*fear of cancer*” in the decreasing order of importance.  Table 6 shows the result of free listing exercise.

**Figure 2:**
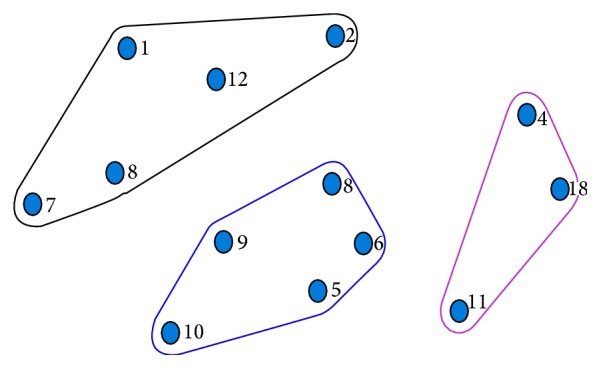
Cluster analysis of perceived reasons of low uptake.

A multidimensional scaling and hierarchical cluster analysis was done with pile sort data to get collective picture of perceived rationale. There were 15 participants in the pile sorting exercise including both health care professionals and laywomen. The results of pile sorting are shown in Figure 2 and Table 7. The three groups which emerged during cluster analysis show concurrence with the findings of the thematic analysis of FGDs. These groups were thus named* (1) information gap leading to fear of unknown, (2) casual attitude*, and* (3) resource constrains and affordability issues.*

**Figure 3:**
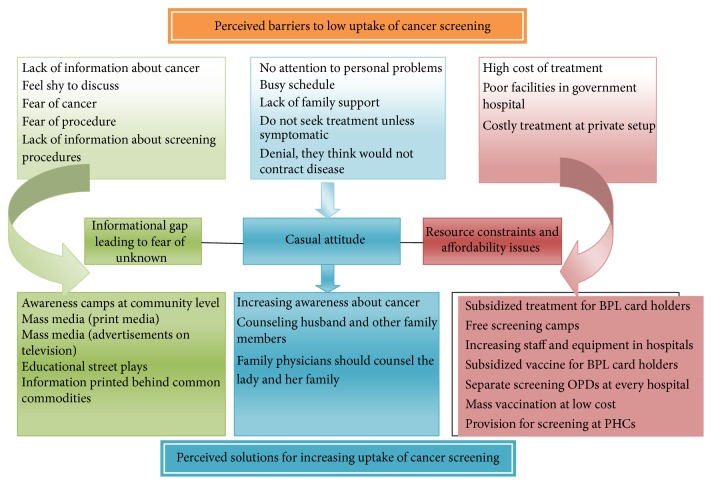
Overview of perceived barriers and their solutions.

With the pile sorted data and its outcome in three major clusters or groups health care professionals and women were approached again to enlist solutions for the barriers separately for each of the three groups. Seventeen participants deliberated separately to generate separate list of solutions for each of the groups. The lists of solutions for each group thus received were free listed again for bringing objectivity and reliability. Tables 8, 9, and 10 show the results of the free listing exercise for each of the three groups of barriers.  Figure 3 shows the overview of the perceived reasons and solutions for low uptake of cervical cancer screening.

## 4. Discussion

The participants of the study were predominantly young with almost 55% of women below 40 years old. Eleven percent of women were illiterate and over 40% of women were graduates or older. According to the Kuppuswamy classification of socioeconomic class the majority seems to be a mix of two classes almost of equal proportion, that is, upper middle class (36%) and upper lower class (39%). This can be explained by the choice of conducting survey, that is, two urban slums and two well planned housing colonies. In the knowledge attitude and practice section of the study which was done through the structured questionnaire, the average knowledge of the section of women interviewed was 18/39 (46.15%) which was below adequate as preset in the questionnaire, that is, 50%. Moreover more than 53.97% of women had their knowledge score below adequate, that is, 19. This population seems to be doing better in terms of baseline knowledge as far as other studies [18, 19] are concerned which measure maximum up to 20% only having adequate knowledge. This observation can be explained by the fact that the questionnaire was designed by the author to measure the knowledge of women sufficient enough to understand the importance of screening and not to gain in-depth knowledge of cervical cancer. Women below 40 years of age have significantly lower mean knowledge scores (16.38 versus 18.46; *p* = 0.05). This difference seems starker when the number of illiterate women is significantly more in the older group (14/170 versus 22/138; *p* < 0.036). There is no significant difference in socioeconomic class among women of the two age groups either to explain the above finding (*p* > 0.05). This could be rather understood by the fact that the knowledge about cervical cancer screening is acquired through real life exposure to health care facilities which older women seem to have more experience with. This is independent of their exposure to formal educational system. Almost 88% of women had heard of cervical cancer but the source of information does not seem to show any predilection to particular method and equally distributed among the sources of information like media, health care professionals, friends, and family. Only 29% of women could identify at least one symptom of cervical cancer and only 24% women knew that it could be caused by sexually transmitted disease. These findings are similar to observations on a cross-sectional study [19] conducted in hospital settings in the same area. Regarding the knowledge of preventive measures and screening, the performance is dismal as shown in previous studies [18, 20, 21]. Although almost half of the women think that cervical cancer is a preventable disease only 34% of women know about cervical cancer screening and only dismal 11 percent of women have ever undergone test [18, 19]. About the vaccine availability 13% of women agreed that they know about the vaccine. This percentage seems to be higher than that shown in other studies [19]. When asked about whether they would like to get vaccinated or their daughters vaccinated only 39.60% of women agreed in the same cost.

The fight against cervical cancer is the fight against human perceptions and misconceptions. Even with the availability of cost effective easily available methods we have not been able to increase the acceptance of cervical cancer screening. Lack of will of the government, the family members, and the women themselves stems from various sociocultural and economic barriers. Qualitative studies have been shown to be helpful in revealing the caveats of human perception and thus devise strategies against them. Qualitative methods have been used now for a long time to understand the barriers against reducing cervical cancer deaths and disease burden. Present study attempted to understand the perceptions of the stakeholders regarding cervical cancer and inquire into possible solutions. Inclusion of both the beneficiaries and health care professionals' perceptions makes this study more comprehensive and widely applicable.

Women strongly perceive that the awareness about cervical cancer is low and needs to be enhanced. They correctly and consistently identify few early symptoms as well as risk factors of cervical cancer. They also are carrying several misconceptions regarding cervical cancer like the fact that not wearing undergarments or prolapsed uterus or decreased menstrual bleeding or surgical abortions can cause cervical cancer. The knowledge about HPV vaccination is very low but women stated that they would like to get their daughters vaccinated if it is available easily at an affordable cost.

The three main themes which emerged from the content analysis about the perceived reasons of low cervical cancer screening are (1) information gap leading to fear of unknown, (2) casual attitude, and (3) resource constrains and affordability issues.

*“Information gap leading to fear of unknown”* seems to be the most strongly felt perception of women which they consider as barrier to proper utilization of screening facility. “*Lack of awareness among females*” and “*illiteracy among females*” were frequently quoted sentences during interview. Other researchers [8, 22] also found this factor to be the most important cause where they have quoted it as “*ignorance about cancer,*” “*cultural constructs/belief about illness,*” and “*low knowledge levels.*” In the free listing exercise women enlisted the causes as* “lack of information about cancer,” “feel shy to discuss,” “fear of cancer,” “fear of procedure,”* and* “lack of information about screening procedure,*” in that order. The perceived solution to these barriers is obviously to indulge in activities which increase awareness. This can be largely summarized into three approaches. (a) Awareness camps in community level, (b) use of electronic or print media, and (c) finally opportunistic counseling by health care professionals.

*“Casual attitude”* is probably the most poorly understood theme of the entire phenomenon. Distinct from lack of awareness, casual attitude stems from issues of gender equality, deprivation of reproductive rights of women, and incapability to take decisions. Deeply ingrained in the society these norms have caused oppression of women in our society to look so natural and devoid of malintention. Women also tend to live with the reality and develop compensatory explanations which mainly appeared in these interviews like “they are busy in daily chores,” “they can never develop cancer,” “they need permission from family,” and so on. Solutions to* casual attitude* as a hindrance to reduced uptake of cervical cancer are multipronged with merging boundaries as the problem itself. Deduction of the solution as suggested by participants can largely be of two types: first is a personalized counseling of the women and family and second is easy access to services. Personalized counseling is in the form of counseling by family physicians, peer help groups, work place counseling sessions, and inclusion of husband and other family in counseling. The second aspect of easy access to services can be done by monitory grants for first screening and free screening in government hospitals and subsidized in private ones.

“*Resource constraints and affordability issues*” are the most widely deliberated issue. “*Long waiting queues and procedural delays,*” “*bad behavior and attitude of doctors and staff,*” “*unreliable diagnosis in government hospitals,*” “*poor satisfaction in government hospitals,”* and “*nonavailability of staff*”* were* the main expressions which we encountered. Other researchers [22–24] named these as “*economic factors*”; “*unfriendly health care services,*” “*large number of patients,*” “*lack of infrastructure and medical supplies,”* and “*lack of policy guidelines*” are among the important ones. The solution to this problem is strengthening the present infrastructure with equipment, use of low cost modern technology, and above all dedicated trained staff who can work at the primary care level. The suggestion included “*training of ASHA workers and staff nurses,*” “*dedicated screening clinics in hospital which are hassle-free and free of cost,*” “*free screening camps,*” “*subsidized vaccination for poor sections of the society,*”* and “development of screening methods which patients themselves can do.”* Most importantly, the need for development of national policy of guideline for cervical cancer screening and infrastructure requirement for the same is felt.

## 5. Conclusion

The perceived reasons for low uptake of cervical cancer screening in the society are largely “low felt need” arising from lack of awareness. In order to reduce the burden of cervical cancer morbidity and mortality due to late diagnosis widespread awareness campaign using multimedia and personalized encouragement by health care professionals at community level and in hospital settings will be effective. Efforts should be made so that the health care facilities reach the masses in an affordable and friendly manner. Subsidized vaccination against HPV and newer self-administered screening tests are areas which should be given more attention in future.

## Figures and Tables

**Table 1:** Demographic date of participants of the survey.

	*N* (%) Total 306
Age (years)	
<30	80 (26.14)
≥30–40	90 (29.41)
≥40–50	70 (22.72)
≥50–60	32 (10.45)
≥60	34 (11.11)
Marital status	
Married	292 (95.42)
Unmarried	14 (4.57)
Educational status	
Professional	8 (2.61)
Graduate	122 (39.86)
SSC	48 (15.68)
HSC	22 (7.14)
Middle school	46 (15.03)
Primary school	26 (8.49)
Illiterate	36 (11.76)
Occupation	
Professional	46 (15.03)
Semiprofessional	46 (15.03)
Clerical, shop owner	34 (11.11)
Skilled worker	10 (3.26)
Semiskilled	38 (12.41)
Unskilled	40 (13.07)
Unemployed	92 (30.06)
Family income (in Rupees)	
≥31,507	54 (17.64)
15,754–31,506	70 (22.87)
11,817–15,753	34 (11.11)
7878–11,816	52 (16.99)
4727–7877	46 (15.05)
1590–4726	44 (14.37)
≤1589	6 (1.97)
Modified Kuppuswamy socioeconomic class	
Upper	40 (13.07)
Upper middle	110 (35.97)
Lower middle	38 (12.41)
Upper lower	116 (37.90)
Lower	2 (0.6)

**Table 2:** Knowledge score regarding cervical cancer.

Knowledge attitude and practice score	
Median score	18 [10–26 (25th and 75th)]
Insufficiently informed (<19)	53.89% (166/308)
Adequately informed (≥19 to <29%)	24.62% (76/308)
Well informed (≥29%)	21.42% (66/308)

**Table 3:** Survey results I.

Baseline knowledge about symptoms and risk factors of cervical cancer	*N* (%) Total 308
Ever heard of cervical cancer (Yes)	87.01% (268/308)
Source of information if heard of cervical cancer	
Health care provider	76 (24.68)
Family or relatives	66 (21.43)
Media	60 (19.48)
Friends	66 (21.43)
Not heard	40 (14.98)
Cervical cancer is the most common female reproductive cancer	172 (55.84)
All women are at risk of cervical cancer?	156 (50.65)
Do you know someone with cervical cancer?	90 (29.22)
Are you aware that the following symptoms are associated with genital cancer? (full marks even if one or more is known)	118 (38.31)
Abnormal vaginal discharge	
Abnormal vaginal bleeding	
Bleeding after sexual intercourse	
Bleeding after menopause	
Are you aware that cervical cancer is caused by a sexually transmitted disease?	74 (24.03)
Are you aware that being very younger at marriage and at the time of birth of first child makes women at risk of cervical cancer?	126 (40.91)

**Table 4:** Survey result II.

Baseline knowledge of available preventive measures	
Are you aware that Cervical cancer can be prevented?	146 (47.40)
Have you heard of cervical cancer testing?	106 (34.42)
Have you heard of center(s) that do cervical cancer testing?	70 (22.73)
*If heard of testing then have you ever undergone cervical cancer testing?*	*34 (11.04)*
Are you aware there is a vaccine which can significantly reduce chances of cervical cancer?	40 (12.99)
The cost of vaccination is Rs. 3000/vaccine and three such vaccines are required. Will you like to get yourself vaccinated if the services are available locally at this cost?	
Yes	122 (39.61)
No	48 (15.58)
Not sure	138 (44.81)

**Table 5:** Thematic analysis of FGD with categorization matrix.

Category	Subcategories	Codes
(1) Baseline knowledge	(1) Perceptions about cervical cancer	Women lack of knowledge about cervical cancer (4)
		It is a very common cancer
		Cervical cancer is an preventable disease
		Every women should be screened
		Very few women undergo test
		Delay in treatment is associated with poor outcome
	(2) Perceptions about the causation of cervical cancer	White discharge is an initial manifestation (5)
		STD of uterus and related structures cause cervical cancer (2)
		Use of condoms can prevent cancer (2)
		Women having multiple sex partners have more risk of cervical cancer (3)
		Irregular periods and heavy periods are associated with cervical cancer (4)
		Pain and itching in private parts can be associated with cervical cancer
		Abdominal mass and weight gain are symptoms of cervical cancer (2)
		Decreased bleeding can be associated with cervical cancer
		Unhygienic practices like use of dirty sanitary napkins cause cervical cancer
		Surgical abortion can cause cervical cancer
		Tobacco consumption is associated with cervical cancer
		Prolapsed uterus can cause cervical cancer
		Not wearing undergarments can be associated with cervical cancer

(2) Perceived reasons for low uptake	(1) Information gap leading to fear of unknown	Lack of awareness about cervical cancer and screening procedures (5)
		Illiteracy among females
		Fear of cancer diagnosis (2)
		Fear of dropping social image (2)
		Fear of procedure (2)
		Talking about sex is a taboo
		No formal sex education by parents results in unsafe sex practices
		No attempt to spread awareness about cervical cancer specially in rural areas (2)
	(2) Casual attitude	They can never develop cervical cancer (3)
		They take personal problems casually
		They are busy in daily chores
		There is lack of family support
		They need permission from family
	(3) Resource constrains and affordability issues	Long waiting queue and procedural delays in government hospitals (6)
		Bad behavior of doctors and staff (2)
		Unreliable diagnosis in government hospitals
		Poor satisfaction in government hospitals
		Nonavailability of doctors and staff in government hospitals (2)
		Being hesitant if doctor is male (4)
		Lack of cheap public transport
		High cost in private hospitals
		Women undergo hysterectomy for trivial complaints
		Preferring to get screening done in camps

(3) Perceived solutions	(1) Increasing awareness	Seminars for lay public (3)
		Television and pamphlets (4)
		Doctors should impart knowledge
		Group discussions among women
		ASHA worker can spread (2) awareness in rural areas
		Camps by Anganwadi workers
	(2) Vaccination	Never heard of it
		Have heard something but not sure
		Would like to accept it is made available
		Would like to get the daughters vaccinated
		Worried about high cost
		Vaccine in for mental satis faction and nothing else

Numbers written after the codes represent the frequency with which the code appeared in FGDs.

**Table 6:** Free listing of perceived reasons of low uptake.

Item number	Perceived reasons	Frequency (%)	Average rank	Salience
1	Lack of information about cancer	88.2	1.6	0.725
2	Feel shy to discuss	64.7	2.09	0.461
3	No attention to personal problems	35.3	1.83	0.301
4	High cost of treatment	23.5	3	0.136
7	Fear of cancer	17.6	2.33	0.113
5	Busy schedule	23.5	3.5	0.09
6	Lack of family support	17.6	2.67	0.078
8	Fear of procedure	11.8	2.5	0.074
10	Denial, they think they would not contract disease, so they do not go for screening	11.8	3	0.059
9	Do not seek treatment unless symptomatic	11.8	4	0.045
11	Poor facilities in government hospital	5.9	6	0.017
12	Lack of information about screening procedures	5.9	5	0.012
13	Costly treatment at private setup	5.9	7	0.008

**Table 7:** Clustering of perceived reasons for low uptake of cervical cancer screening.

	Reasons of low uptake	Groups
1	Lack of information about cancer	Informational gap leading to fear of unknown
2	Feel shy to discuss	
7	Fear of cancer	
8	Fear of procedure	
12	Lack of information about screening procedures	

3	No attention to personal problems	Casual attitude
5	Busy schedule	
6	Lack of family support	
9	Do not seek treatment unless symptomatic	
10	Denial, they think they would not contract disease	

4	High cost of treatment	Resource constraints and affordability issues
11	Poor facilities in government hospital	
13	Costly treatment at private setup	

**Table 8:** Perceived solutions for “*information gap leading to fear of the unknown.*”

Perceived solutions	Frequency (%)	Average rank	Salience
(i) Awareness camps at community level	80	1.25	0.742
(ii) Mass media (print media)	53.3	2.38	0.328
(iii) Mass media (advertisements on television)	40	2.17	0.267
(iv) Educational street plays	20	2	0.139
(v) Information printed behind common commodities	20	2.33	0.122
(vi) Organizing group discussions	20	3.33	0.069
(vii) Educational camps in schools	13.3	2	0.103
(viii) School students led to home to home awareness	6.7	5	0.013
(ix) Counseling centers at community level	6.7	2	0.033
(x) Announcements at public places	6.7	4	0.017
(xi) Free screening at gynecology OPD	6.7	2	0.033
(xii) Doctors should educate about screening	6.7	3	0.033
(xiii) CHW led to home to home spread of awareness	6.7	4	0.027

**Table 9:** Perceived solutions for “*casual attitude.*”

Perceived solutions	Frequency (%)	Average rank	Salience
(i) Increasing awareness about cancer	73.3	1.27	0.65
(ii) Counseling husband and other family members	40	1.5	0.322
(iii) Family physicians should counsel the lady and family	13.3	1.5	0.111
(iv) Group counseling at work places	6.7	3	0.033
(v) Government can offer monetary grant for first screening	6.7	4	0.017
(vi) Acknowledging that every lady is susceptible	6.7	2	0.044
(vii) Door to door provision of screening and vaccination	6.7	1	0.067
(viii) Free screening at OBGY OPD	6.7	3	0.022
(ix) Educating young females and encouraging them to spread awareness among their relatives and friends	6.7	1	0.067

**Table 10:** Perceived solutions for “resource constrains and affordability issues.”

Perceived solutions	Frequency (%)	Average rank	Salience
(i) Subsidized treatment for BPL card holders	40	1.5	0.328
(ii) Free screening camps	26.7	1.5	0.2
(iii) Increasing staff and equipment in government hospitals	26.7	2	0.189
(iv) Subsidized vaccine for BPL card holders	20	1	0.2
(v) Separate screening OPDs at every government hospital	13.3	3	0.044
(vi) Mass vaccination at low cost	13.3	1.5	0.1
(vii) Provision for screening at primary health centers	13.3	1	0.133
(viii) Training nurses especially for Pap smear	13.3	3.5	0.039
(ix) ASHA and USHA workers trained to do screening at village	6.7	1	0.067
(x) Self-screening kits can be developed	6.7	2	0.044
(xi) Allowing payment of vaccine in installments	6.7	2	0.033
(xii) Discounted treatment by pharmaceutical companies	6.7	2	0.033
(xiii) Government schemes to promote cancer screening at every level	6.7	2	0.044
(xiv) Health camps aiming at high-risk population	6.7	1	0.067
(xv) Certain days of the year should be allotted for cervical cancer screening on a mass scale (like pulse polio)	6.7	3	0.022
(xvi) Government policies for cost control in private hospitals	6.7	3	0.022
(xvii) Educating about low cost of screening and treatment at government hospitals	6.7	1	0.067

## References

[1] Institute for Health Metrics and Evaluation (2011). *The Challenge Ahead: Progress in Breast and Cervical Cancer*.

[2] Varughese J., Richman S. (2010). Cancer care inequity for women in resource-poor countries. *Reviews in Obstetrics and Gynecology*.

[3] Shafi M. I., Earl H. M., Tan L. T. (2010). *Gynaecological Oncology*.

[4] Goldie S. J., Levin C., Mosqueira-Lovón N. R. (2012). Health and economic impact of human papillomavirus 16 and 18 vaccination of preadolescent girls and cervical cancer screening of adult women in Peru. *Revista Panamericana de Salud Pública*.

[5] Vallikad E. (2006). Cervical cancer: the Indian perspective. *International Journal of Gynecology and Obstetrics*.

[6] Jacob M., Mawar N., Menezes L. (2010). Assessing the environment for introduction of human papillomavirus vaccine in India. *Open Vaccine Journal*.

[7] Madhivanan P., Krupp K., Yashodha M. N., Marlow L., Klausner J. D., Reingold A. L. (2009). Attitudes toward HPV vaccination among parents of adolescent girls in Mysore, India. *Vaccine*.

[8] Fort V. K., Makin M. S., Siegler A. J., Ault K., Rochat R. (2011). Barriers to cervical cancer screening in Mulanje, Malawi: a qualitative study. *Patient Preference and Adherence*.

[9] (2005). *National Cancer Control Programme Guidelines*.

[10] (2005). *Guidelines for Cervical Cancer Screening Programme*.

[11] Elo S., Kyngäs H. (2008). The qualitative content analysis process. *Journal of Advanced Nursing*.

[12] Hudelson P. M. (1994). *Qualitative Research for Health Programmes*.

[13] Marshall M. N. (1996). Sampling for qualitative research. *Family Practice*.

[14] Schrauf R. W., Sanchez J. (2004). The preponderance of negative emotion words in the emotion lexicon: a cross-generational and cross-linguistic study. *Journal of Multilingual and Multicultural Development*.

[15] Hsieh H.-F., Shannon S. E. (2005). Three approaches to qualitative content analysis. *Qualitative Health Research*.

[16] Graneheim U. H., Lundman B. (2004). Qualitative content analysis in nursing research: concepts, procedures and measures to achieve trustworthiness. *Nurse Education Today*.

[17] Borgatti S. (1998). *ANTHROPAC (Computer Program)*.

[18] Shrestha J., Saha R., Tripathi N. (2013). Knowledge, attitude and practice regarding cervical cancer screening amongst women visiting tertiary centre in Kathmandu, Nepal. *Nepal Journal of Medical Sciences*.

[19] Bansal A. B., Pakhare A. P., Kapoor N., Mehrotra R., Kokane A. M. (2015). Knowledge, attitude, and practices related to cervical cancer among adult women: a hospital-based cross-sectional study. *Journal of Natural Science, Biology and Medicine*.

[20] Singh E., Seth S., Rani V., Srivastava D. K. (2012). Awareness of cervical cancer screening among nursing staff in a tertiary institution of rural India. *Journal of Gynecologic Oncology*.

[21] Singh M., Ranjan R., Das B., Gupta K. (2014). Knowledge, attitude and practice of cervical cancer screening in women visiting a tertiary care hospital of Delhi. *Indian Journal of Cancer*.

[22] Mutyaba T., Faxelid E., Mirembe F., Weiderpass E. (2007). Influences on uptake of reproductive health services in Nsangi community of Uganda and their implications for cervical cancer screening. *Reproductive Health*.

[23] Chirenje Z. M., Rusakaniko S., Kirumbi L. (2001). Situation analysis for cervical cancer diagnosis and treatment in east, central and southern African countries. *Bulletin of the World Health Organization*.

[24] Kivuti-Bitok L. W., Pokhariyal G. P., Abdul R., McDonnell G. (2013). An exploration of opportunities and challenges facing cervical cancer managers in Kenya. *BMC Research Notes*.

